# Amino Acid Composition of Breast Milk from Urban Chinese Mothers

**DOI:** 10.3390/nu8100606

**Published:** 2016-09-28

**Authors:** Clara L. Garcia-Rodenas, Michael Affolter, Gerard Vinyes-Pares, Carlos A. De Castro, Leonidas G. Karagounis, Yumei Zhang, Peiyu Wang, Sagar K. Thakkar

**Affiliations:** 1 Nestlé Research Center, Nestec Ltd., Lausanne 1000, Switzerland; michael.affolter@rdls.nestle.com (M.A.); carlosantonio.decastro@rdls.nestle.com (C.A.D.C.); leonidas.karagounis@rdls.nestle.com (L.G.K.); sagar.thakkar@rdls.nestle.com (S.K.T.); 2 Nestlé Health Sciences, Nestec Ltd., Epalinges 1066, Switzerland; gerard.vinyespares@nestle.com; 3 Department of Nutrition and Food Hygiene, School of Public Health, Peking University, Beijing 100191, China; zhangyumei@hsc.pku.edu.cn; 4 Department of Social Medicine and Health Education, School of Public Health, Peking University, Beijing 100191, China; wpeiyu@bjmu.edu.cn

**Keywords:** breast milk, amino acids, lactation period, cross-sectional study

## Abstract

Human breast milk (BM) amino acid (AA) composition may be impacted by lactation stage or factors related to geographical location. The present cross-sectional study is aimed at assessing the temporal changes of BMAA over lactation stages in a large cohort of urban mothers in China. Four hundred fifty BM samples, collected in three Chinese cities covering eight months of lactation were analyzed for free (FAA) and total (TAA) AA by o-phthalaldehyde/ fluorenylmethylchloroformate (OPA/FMOC) derivatization. Concentrations and changes over lactation were aligned with previous reports. Both the sum and the individual TAA values significantly decreased during the first periods of lactation and then generally leveled off. Leucine and methionine were respectively the most and the least abundant indispensable amino acids across all the lactation stages, whereas glutamic acid + glutamine (Glx) was the most and cystine the least abundant dispensable AA. The contribution of FAA to TAA levels was less than 2%, except for free Glx, which was the most abundant FAA. In conclusion, the AA composition of the milk from our cohort of urban Chinese mothers was comparable to previous studies conducted in other parts of the world, suggesting that this is an evolutionary conserved trait largely independent of geographical, ethnic, or dietary factors.

## 1. Introduction

Evolution has shaped the composition of breast milk to ensure optimal development of healthy term offspring. However, breast milk composition is not constant and appears to be affected by multiple factors, including lactation stage, mothers’ genetic background and diet, gestational age at delivery, or geographical location [1].

Breast milk protein is a key nutrient supporting body growth and organ development during the first few months of life by providing nitrogen and indispensable amino acids (IAA) required for body protein building and by stimulating the secretion of growth-promoting hormones (i.e., insulin, insulin-like growth factor (1-IGF1)). Potent insulinotropic amino acids such as the branched chain amino acids—Leucine, Lysine, and Threonine—can be particularly important in this context. However, emerging evidence suggests that the relatively low levels of protein and insulinotropic amino acids in breast milk may be protective against the development of metabolic disorders later in infant life [2]. Because body weight, body composition, growth rate, and volume of milk intake are known to change with an infant’s age [3,4], infant requirements in terms of both protein and individual amino acid composition also varies along the different stages of lactation [3].

Most amino acids in breast milk are found as constituents of protein chains, but there is also a certain amount of free amino acids (FAA), which usually account for less than 10% of the total amino acid (TAA) levels [5,6]. Although still poorly explored, emerging evidence suggests specific physiological roles of the FAA fraction, such as appetite control [7]. Many studies have analyzed the TAA content in human milk, but they often characterize a limited number of samples, do not account for the important lactation-stage associated changes, or both. The number of studies on FAA is even more limited. In their systematic review of breast milk amino acid composition studies from different continents, Zhang et al. [8] report geographical differences in the content of some TAA and FAA, although data from some regions of the world is relatively limited. In particular, studies looking at breast milk protein quality in China are scarce, with only two small studies reporting on the average TAA composition of one to six months [9] and 7–180 days [10] postpartum milk. To our knowledge, no data on TAA and FAA content in milk from Chinese mothers along lactation is available to date.

The objective of this cross-sectional study was to assess the temporal changes of FAA and TAA in milk secreted during the different stages of lactation in a large cohort of Chinese mothers from three different cities in urban China.

## 2. Materials and Methods

### 2.1. Subjects

This study was part of the Maternal, Infant and Nutrition Growth study (MING), a cross-sectional study designed to investigate the dietary and nutritional status of pregnant women, lactating mothers, and young children aged from birth up to three years living in urban areas of China [11]. In addition, the human milk composition of the lactating mothers was characterized. The study was conducted between October 2011 and February 2012. A multi-stage milk sampling from lactating mothers in three cities (Beijing, Suzhou, and Guangzhou) was performed for breast milk characterization. In each city, two hospitals with maternal and child care units were randomly selected; at each site, mothers at lactation periods from 0 to 240 days were randomly selected based on child registration information. Subjects included in the 0–5-day period were recruited at the hospital, whereas the other subjects were invited by telephone to join the study; if participation was dismissed, a replacement was found. Response rate was 52%. Recruitment, milk collection, and baseline data collection were completed on separate days.

Stratified milk sampling of 540 lactating mothers in six lactation periods of 0 to 4, 5 to 11, and 12 to 30 days, and 1–2, 2–4, and 4–8 months, was obtained in the MING study. Nevertheless, only 450 milk samples were analyzed in the amino acid study, as the 0- to 4-day stage could not be included due to the limited volume of milk collected during this period.

Eligibility criteria included women between 18 and 45 years of age giving birth to a single, healthy, full-term infant and exclusively breastfeeding until at least 4 months after birth. Exclusion criteria included gestational diabetes, hypertension, cardiac diseases, acute communicable diseases, and postpartum depression. Lactating women who had nipple or lacteal gland diseases, who had been receiving hormonal therapy during the three months preceding recruitment, or who had insufficient skills to understand study questionnaires were also excluded.

The study was conducted according to the guidelines in the Declaration of Helsinki. All of the procedures involving human subjects were approved by the Medical Ethics Research Board of Peking University (No. IRB00001052-11042). Written informed consent was obtained from all subjects participating in the study. The study was registered at ClinicalTrials.gov (NCT01971671)

### 2.2. Data Collection

All subjects responded to a general questionnaire including socio-economic and lifestyle aspects of the mother. The self-reported weight at delivery, the number of gestational weeks at delivery, and the delivery method were also recorded. Additionally, a physical examination (height, weight, mid-arm circumference, blood pressure, and hemoglobin levels) was also carried out.

Data collection was done through face-to-face interviews on the day of milk sample collection. The infant’s date of birth and gender information was collected retrospectively by phone interview.

### 2.3. Sample Collection

Breast milk sampling was standardized for all subjects and performed with an electric pump (Horigen HNR/X-2108ZB, Xinhe Electrical Apparatuses Co., Ltd., Beijing, China). Samples were collected at the second feeding in the morning (9–11 a.m.) to avoid circadian influence on the outcomes. Single full breast was emptied, and an aliquot of 40 mL was secured for characterization purposes. The rest of the milk was returned to the mother for infant feeding. One-milliliter aliquots of each sample were transported on dry ice to a laboratory and stored at −80 °C until further analysis.

### 2.4. Amino Acid Analysis

All samples were analyzed by Eurofins Technology Service (Suzhou) Co. Ltd., Suzhou, China.

TAA content was determined according to a validated o-phthalaldehyde/fluorenylmethylchloroformate (OPA/FMOC) derivatization procedure described by Blankenship et al. [12]. Briefly, protein-bound amino acids were converted to the free state by acid hydrolysis in 6 M of hydrochloric acid at 110 °C for 22 h with a phenol antioxidant in the absence of oxygen. The digests were derivatized with ortho-phthalaldehyde (OPA), mecaptopropionic acid (MCP), and 9-fluorenylmethyl chloroformate (FMOC-Cl) under alkaline conditions prior to injection. Separation and quantification of the amino acid derivatives were performed by high-performance liquid chromatography HPLC with a UV/diode array and fluorescence detection. The limit of detection (LOD) was 1 mg/100 g and the limit of quantification (LOQ) was 5 mg/100 g. Average repeatability was 12%, and reproducibility between duplicate determinations was 18% for the 18 measured amino acids with recoveries ranging from 64.9% to 129.6%.

FAA content was determined according to the same OPA/FMOC method, but without the acid hydrolysis step. All samples were analyzed in duplicate.

### 2.5. Statistical Analysis

Multiple linear regression was applied to analyze the effect of the lactation period on the levels of TAA and FAA. This model was adjusted for the effects of maternal age and body mass index (BMI), infant gender, mode of delivery, and geographical location. Comparisons were made regarding each subsequent lactation period (5–11 days vs. 12–30 days, 12–30 days vs. 1–2 months, 1–2 months vs. 2–4 months, and 2–4 months vs. 4–8 months) by calculating contrast estimates produced by the model.

For the socio-demographic and anthropometric data, analysis of variance was applied for the continuous variable in question and the lactation period in order to check if there was at least 1 period that was different than the others. For factor variables, an independence test was performed in order to detect differences in distribution among the different period.

All statistical analyses were performed with the statistical software R (version 3.0.1; R Foundation, Vienna, Austria).

## 3. Results

### 3.1. Subject Characteristics

**Figure 1:**
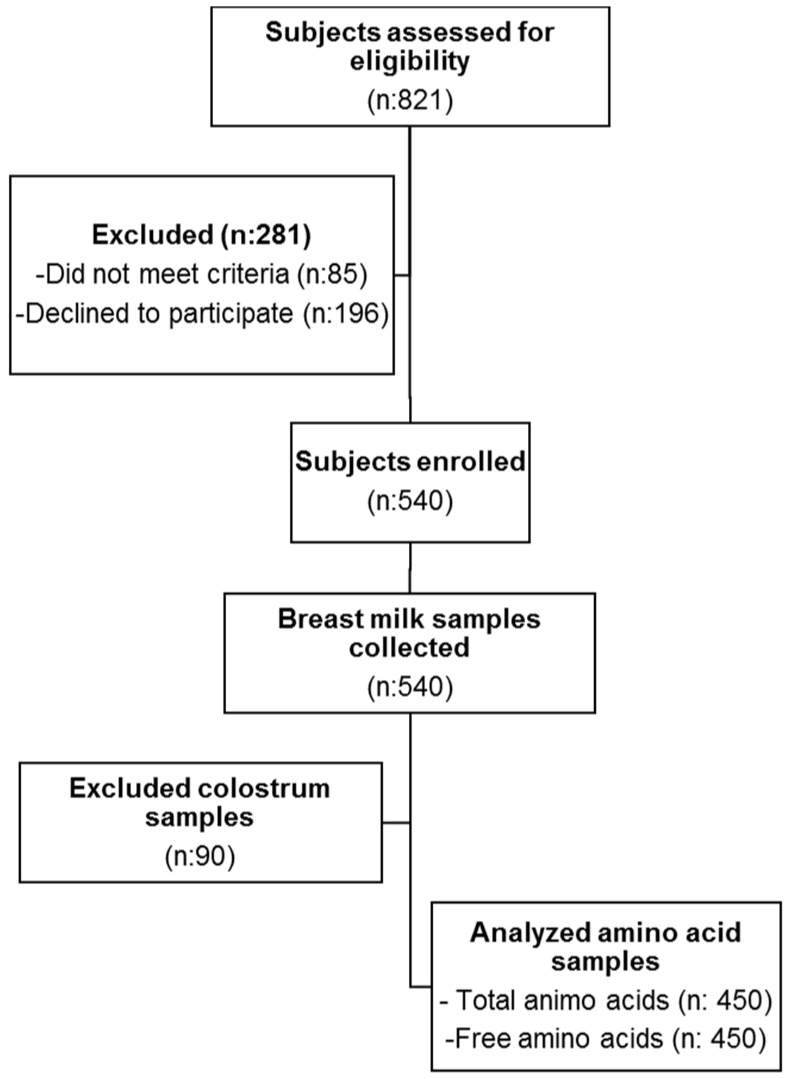
Study flow chart.

In this cross-sectional study, TAA and FAA were quantified in 450 breast milk samples collected at different stages from early to late lactation in healthy urban Chinese women. The recruitment flowchart from eligibility to sample analysis is illustrated in Figure 1.

Subject demographics and anthropometry are described in Table 1. Maternal age, weight, BMI, and mode of delivery were significantly different among the lactation stage cohorts. No other significant differences were observed in maternal and infant characteristics analyzed.

### 3.2. Total Amino Acids

The levels of TAA were compared across different lactation stages after adjusting for maternal age and BMI as well as for mode of delivery, infant gender, and geographical location.

**Figure 2:**
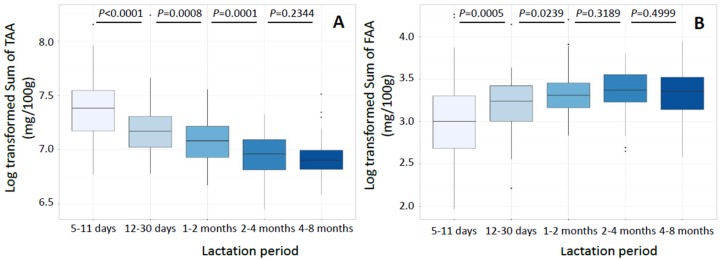
Box plot of the log-transformed sum of total (TAA, (**A**)) and of free (FAA, (**B**)) amino acids in milk from the different lactation periods. *n* = 90 milk samples per lactation period. Statistically significant differences between two periods were set at *p* < 0.05.

The sum of TAA in milk samples significantly decreased with increasing lactation stage until the 2–4-month milk, which did not differ significantly from that at 4–8 months (Figure 2A). Median values ranged between 1608 mg/100 g and 1053 mg/100 g in the 5–11-day and the 2–4-month samples, respectively.

Concentrations of total IAA are reported in Table 2. Leucine and methionine were respectively the most and the least abundant IAA in our sample set across all the lactation stages. The levels of all IAA were highest in the early milk samples and then decreased with increasing lactation period until 2–4 months. Some differences of lower magnitude were still perceived between the two latest lactation stages; in particular, the levels of Leucine, lysine, and methionine were higher and the levels of histidine and phenylalanine were lower in 2–4-month than in 4–8-month milk.

Regarding dispensable amino acids (DAA) (Table 2) Glx (sum of glutamic acid + glutamine) was the most and cystine the least abundant amino acids. Again, the highest concentration for all DAA was recorded in the earliest milk (i.e., 5–11 days), and a subsequent decrease was observed in most DAA across the intermediate time points until reaching similar levels at the two latest lactation stages. In contrast, stable levels were observed for Glx and Asx (sum of aspartic acid + asparagine) between the 12–30-day and 2–4-month milk. A further decrease occurred in the 4–8-month samples.

### 3.3. Free Amino Acids

The levels of FAA were compared across the different lactation stages after adjusting for maternal age and BMI as well as for mode of delivery, infant gender, and geographical location.

In contrast to TAA, the sum of the individual FAA content was lower in the first compared with the latest lactation stages, with median values ranging between 20.1 mg/100 g of milk at 5–11 days and 29.0 mg/100 g of milk at 2–4 months (Figure 2B).

Concentrations of the individual FAA are reported in Table 3. Glx was the most abundant in FAA across the data set, and its concentration was higher in mature milk than in early-stage milk. In the latest lactation stages, it contributed up to more than 70% of the FAA mass. Similarly to Glx, levels of alanine, cystine, glycine, and serine were lowest in the early-stage milk. Opposite changes were observed in free IAA, of which highest concentrations were generally found in early-stage samples. The only exception was threonine, which remained stable across the lactation periods.

The contribution of FAA to TAA levels was less than 2% for most amino acids studied. A major exception was free Glx, which, on average, contributed to around 8% of the total Glx concentration. The ratio of free to total Glx gradually increased across the increasing lactation stages, from less than 5% at 5–11 days up to more than 10% at 4–8 months.

Of note is that compared with the TAA (Table 2) inter-individual variability in FAA values was very high (Table 3).

## 4. Discussion

Amino acids are an essential component in infant nutrition, and their levels in breast milk are believed to be optimal to support healthy growth during the first months of life. Because of this, the amino acid intake from human milk is considered to match infant requirements, and the breast milk amino acid content is used to estimate the protein quality and quantity in breast milk substitutes [3]. Therefore, a reliable evaluation of the amino acid composition in breast milk is important.

The concentration of the sum of TAA—a good proxy of the true protein content—at the different lactation stages was, in our samples, remarkably similar to that reported in transitional, mature, and late milk in a recent systematic review of studies from Africa, Asia, Europe, and North America [8]. A limitation in our study, however, is the lack of colostrum samples. This said, although colostrum is important for the protection of the neonate, the amount of amino acids provided by the colostrum protein is likely limited due to the low secreted volumes as well as to the relatively low digestibility of the major colostrum proteins [13]. Similar to the results reported in the systematic review by Zhang et al. [8], the sum of TAA was greater in the early stage of lactation, slowly declining in concentration as lactation progressed, reaching generally stable levels after 2–4 months. These changes are consistent with the protein content results of the MING study reported elsewhere [11] and with the well-known evolution of the protein content in breast milk, i.e., high during the early lactation stages, and sharply decreasing during the transitional milk period to level off in mature milk [13]. It has been proposed that these changes in the amino acid content of the milk match the infant requirements for growth, which is fast during the neonatal period sharply decreasing during the first months of life [14]. Declining protein concentrations may also prevent amino acid overfeeding as milk volume intake per unit body weight increases along lactation [3,4]. Of note, protein concentration in human milk is remarkably low compared with that from most other mammals. Low protein intake during infancy is believed to protect the individual against obesity and metabolic disease later in life, possibly related to optimal appetite and hormonal programming [15].

The levels of individual TAA’s in the different lactation stages of the MING cohort were also close to those reported by Zhang et al. [8]. The only amino acid showing a consistently lower value in our samples was cystine. However, cystine is known to be particularly sensitive to the acid treatment used for protein hydrolysis in our samples [16], and the cystine levels that we report here may underestimate the real values.

Globally, the individual TAA levels showed similar temporal patterns as the sum of TAA. However, whereas a strong decrease between early, transitional, and mature milk was observed for some amino acids such as methionine, cystine, and glycine, this drop was less substantial for Glx levels, which were comparable in transitional and mature milk. This observation is consistent with the fact that besides the variations in protein content, changes in the quality of the protein occur along lactation. Specifically, a significant decline in the concentration of the sulfur amino acid-rich and glycine-rich whey proteins but stable levels of the Glx-rich casein were found in our milk samples [17] and are usually reported [18], resulting in a whey to casein ratio that increases throughout lactation [13] and, as observed in our samples, in an evolving amino acid profile.

As expected from previous reports [6], the contribution of FAA to the TAA mass was less than 3% in all lactation periods. Because of this, the contribution of the FAA to the nutritional requirements of the infant is expected to be low. The physiological importance of FAA for infants is not yet well understood. It has been proposed that FAA are more rapidly absorbed, leading to accelerated appearance in the systemic circulation and thus reaching the peripheral organs faster than the protein-bound amino acids [6,8]. However, to our knowledge, the absorption kinetics of free and protein-bound amino acids in breast milk has never been compared, and the physiological relevance of a potentially faster delivery to the peripheral tissue of the small FAA load delivered by milk remains speculative.

Free Glx was very abundant in our samples, contributing to around 70% of the FAA mass and reaching up to 10% of the total Glx levels in the later lactation periods. Similar observations have also previously been reported where both glutamic acid and glutamine were shown to be the most abundant FAA in human milk throughout the first trimester of lactation [19]. Specifically, the authors of [19] reported a 2.5- and 20-fold increase in glutamic acid and glutamine FAA concentrations, respectively, with progressing lactation. It should be noted that, similar to our findings, these FAAs represented more than 50% of total FAAs at three months [19]. Total Glx was also the most concentrated TAA in our samples, suggesting an important role of this amino acid on the mammary gland metabolism, on infant nutrition, or both, despite the fact that glutamic acid and glutamine are considered DAA that can be synthesized by the body [20]. More specifically, the transamination of glutamic acid by the mucosal intestinal cells yields alanine, which enters the gluconeogenic pathway. In addition, both glutamic acid and glutamine from the lumen act as major energy substrates for the intestinal cells [21,22]. In the neonatal pig for instance, the gastrointestinal tract uses dietary glutamine and glutamate as its key respiratory fuel. In humans, trials with very low birth weight infants and critically ill adult patients highlight the central role of glutamic acid and glutamine in protecting intestinal growth and integrity [23,24,25], therefore suggesting glutamic acid and glutamine as important molecules in milk for the immature infant gut. More recent results from Ventura et al. [7] also suggest a role of Glutamate on the satiety status of the lactating infant.

Similar to Glx, free alanine, cystine, glycine, and serine also increased along the lactation periods in our samples. Intriguingly, similar findings were reported in the systematic review by Zhang et al. [8], indicating a consistent pattern in stage-associated changes independent of ethnic or geographic factors. This consistency is outstanding in light of the important inter-individual FAA variability in our study, the inter-study variability observed in the report by Zhang et al., and even the intra-individual changes reported by others in transitional and mature breast milk [26]. However, the physiological significance of the concentration rise of these DAA through lactation is not clear. Furthermore, understanding the physiological relevance of the increased inter-individual variability observed in FAA needs to be elucidated.

An important limitation in our study is that our analytical method did not permit to quantify the concentration of tryptophan. Yet the IAA tryptophan is usually the limiting amino acid in infant formula; thus, its concentration in breast milk is often used to estimate the protein quality and to adjust the level of the protein in the formula. Another limitation is the cross-sectional nature of the study that weakens the conclusions related to the stage-driven changes, which would have been best assessed by a longitudinal design. However, our statistical model adjusted for the maternal and infant baseline factors that were known or suspected to impact milk nutrient composition [1], including maternal weight and mode of delivery, which differed between the lactation period cohorts. Our results are also reinforced by the fact that they were remarkably consistent with those previously published.

## 5. Conclusions

In conclusion, the amino acid composition of the milk from our cohort of urban Chinese mothers was comparable to human milk data from previously reported studies carried out in other parts of the world, suggesting that amino acid composition in breast milk is an evolutionary conserved trait largely independent of geographical, ethnical, or dietary factors.

## Figures and Tables

**Table 1:** Maternal and infant characteristics.

	Lactation Period					
	5–11 Days	12–30 Days	1–2 Months	2–4 Months	4–8 Months	*p* Value
	(*n* = 90)	(*n* = 90)	(*n* = 90)	(*n* = 90)	(*n* = 90)	
**MOTHER**						
Age (years), Mean (SD)	27 (4)	27 (3)	28 (4)	27 (4)	26 (4)	0.005
Height (cm), Mean (SD)	160 (4)	160 (5)	161 (5)	161 (5)	159 (5)	0.102
Weight (kg), Mean (SD)	60.7 (8.7)	60.8 (7.9)	61.9 (8.9)	58.4 (8.3)	56.2 (8.1)	<0.001
BMI (kg/m^2^), Mean (SD)	23.7 (3.2)	23.7 (3.0)	23.9 (3.1)	22.5 (2.9)	22.2 (3.1)	<0.001
Gestational weight gain (kg), Mean (SD)	16.7 (7.4)	16.2 (6.0)	15.9 (5.7)	15.9 (5.9)	14.9 (7.6)	0.419
Postpartum weight loss (kg), Mean (SD)	9.1 (6.1)	8.6 (5.3)	9.8 (4.0)	10.0 (6.2)	10.6 (5.9)	0.119
Non-Smoker, *n* (%)	90 (100)	89 (99)	90 (100)	86 (98)	89 (100)	0.176
Cesarean delivery, *n* (%)	39 (42)	43 (48)	53 (59)	35 (39)	35 (38)	0.004
Household income (RMB/month)						
<2000 RMB, *n* (%)	20 (22)	17 (19)	24 (27)	26 (29)	31 (34)	
2000–4000 RMB, *n* (%)	37 (41)	45 (50)	41 (46)	40 (44)	41 (46)	
>4000 RMB, *n* (%)	30 (33)	22 (24)	23 (26)	22 (24)	18 (20)	
Unknown, *n* (%)	1 (1)	6 (7)	2 (2)	0 (0)	0 (0)	0.206
**INFANT**						
Males, *n* (%)	51 (57)	48 (53)	48 (53)	54 (60)	43 (48)	0.865
Gestational age at birth (weeks), Mean (SD)	39.3 (1.2)	39.2 (1.3)	39.2 (1.6)	39.4 (1.3)	39.5 (1.5)	0.684

**Table 2:** Total amino acid content (mg/100 g) of milk from the different lactation periods.

	Lactation Period				
	5–11 Days	12–30 Days	1–2 Months	2–4 Months	4–8 Months
**IAA ^†^**					
Histidine	51.2 (19.9)	44.5 ^§^ (14.1)	36.5 ^§^ (12.6)	34.9 ^§^ (7.2)	25.0 ^§^ (6.8)
Isoleucine	81.0 (23.4)	71.6 ^§^ (15.4)	64.6 ^§^ (16.8)	54.0 ^§^ (11.6)	53.8 (10.7)
Leucine	153.7 (63.2)	133.7 ^§^ (35.1)	130.3 (33.5)	108.1 ^§^ (24.9)	122.6 ^§^ (38.8)
Lysine	112.0 (31.0)	93.8 ^§^ (23.1)	78.8 ^§^ (18.9)	63.4 ^§^ (13.1)	67.9 ^§^ (13.1)
Methionine	21.8 (11.7)	16.7 ^§^ (6.6)	13.0 ^§^ (9.0)	9.2 ^§^ (6.1)	11.8 ^§^ (7.1)
Phenylalanine	64.4 (35.9)	52.4 ^§^ (18.3)	40.4 ^§^ (13.6)	37.6 ^§^ (10.8)	28.4 ^§^ (9.0)
Threonine	85.1 (28.1)	66.9 ^§^ (14.6)	58.0 ^§^ (13.3)	50.0 ^§^ (8.7)	48.6 (11.3)
Valine	97.9 (34.3)	81.1 ^§^ (16.7)	72.1 ^§^ (21.0)	59.7 ^§^ (16.0)	60.9 (12.7)
**DAA ^†^**					
Alanine	70.9 (23.0)	55.9 ^§^ (14.3)	45.9 ^§^ (15.7)	38.7 ^§^ (10.9)	38.6 (9.1)
Arginine	106.5 (36.6)	90.8 ^§^ (22.8)	77.0 ^§^ (24.5)	64.6 ^§^ (21.3)	65.3 (16.7)
Asx ^‡^	132.9 (84.1)	115.5 ^§^ (54.4)	106.9 (40.0)	97.2 (56.8)	83.8 ^§^ (24.6)
Cystine	25.4 (12.5)	17.7 ^§^ (6.3)	12.5 ^§^ (5.2)	12.3 (3.4)	9.9 ^§^ (5.5)
Glx ^‡^	248.1 (193.7)	220.1 ^§^ (92.4)	216.3 (59.3)	188.6 (105.2)	182.8 ^§^ (30.8)
Glycine	46.3 (15.2)	34.5 ^§^ (9.7)	27.6 ^§^ (10.5)	23.6 ^§^ (7.0)	23.5 (6.8)
Proline	140.2 (42.4)	117.7 ^§^ (26.5)	110.6 ^§^ (25.4)	95.3 ^§^ (20.9)	94.5 (17.2)
Serine	77.8 (27.0)	59.0 ^§^ (14.1)	47.9 ^§^ (9.8)	42.9 ^§^ (8.1)	41.7 (8.0)
Tyrosine	72.5 (30.4)	57.7 ^§^ (14.1)	44.1 ^§^ (19.5)	41.4 (13.9)	37.1 ^§^ (10.3)
**SUM**	1608.3 (589.5)	1296.5 ^§^ (368.4)	1188.1 ^§^ (341.7)	1053.2 ^§^ (291.9)	992.4 (175.9)

^†^ IAA = indispensable amino acids; DAA = dispensable amino acid; ^‡^ Asx = sum of aspartic acid + asparagine; Glx = sum of glutamic acid + glutamine. Medians (inter-quartile ranges) of *n* = 90 samples per lactation period are shown. A median with a “^§^” superscript is significantly different from the median of the previous lactation period (*p* < 0.05).

**Table 3:** Free AA content (mg/100 g) of milk from the different lactation periods.

	Lactation Period				
	5–11 Days	12–30 Days	1–2 Months	2–4 Months	4–8 Months
**IAA ^†^**					
Histidine	0.29 (0.21)	0.42 ^§^ (0.23)	0.33 ^§^ (0.15)	0.33 (0.19)	0.28 (0.10)
Isoleucine	0.17 (0.11)	0.19 (0.13)	0.13 ^§^ (0.10)	0.13 (0.07)	0.15 ^§^ (0.07)
Leucine	0.33 (0.20)	0.4 (0.2)	0.34 ^§^ (0.14)	0.33 (0.14)	0.34 (0.15)
Lysine	0.61 (0.51)	0.56 ^§^ (0.28)	0.46 ^§^ (0.20)	0.42 ^§^ (0.23)	0.54 ^§^ (0.28)
Methionine	0.11 (0.07)	0.13 (0.13)	0.10 ^§^ (0.07)	0.07 ^§^ (0.06)	0.12 ^§^ (0.05)
Phenylalanine	0.31 (0.17)	0.40 (0.17)	0.32 ^§^ (0.17)	0.33 (0.17)	0.30 (0.12)
Threonine	0.69 (0.38)	0.69 (0.36)	0.70 (0.36)	0.78 (0.34)	0.85 (0.38)
Valine	0.58 (0.27)	0.70 ^§^ (0.30)	0.61 ^§^ (0.21)	0.59 (0.21)	0.59 (0.18)
**DAA ^†^**					
Alanine	1.26 (0.81)	1.75 ^§^ (0.79)	2.07 ^§^ (0.67)	1.93 (0.68)	1.85 (0.46)
Arginine	0.46 (0.49)	0.42 ^§^ (0.28)	0.25 ^§^ (0.22)	0.25 (0.19)	0.25 (0.13)
Asx ^‡^	0.47 (0.36)	0.52 (0.30)	0.54 (0.35)	0.55 (0.38)	0.58 (0.40)
Cystine	0.32 (0.13)	0.49 ^§^ (0.21)	0.46 (0.17)	0.49 (0.21)	0.50 (0.15)
Glx ^‡^	10.89 (9.89)	15.09 ^§^ (8.74)	18.03 ^§^ (7.17)	20.22 ^§^ (7.28)	19.36 (8.07)
Glycine	0.51 (0.29)	0.62 ^§^ (0.23)	0.68 ^§^ (0.25)	0.64 (0.28)	0.76 ^§^ (0.28)
Proline	0.56 (0.33)	0.40 ^§^ (0.28)	0.54 (0.29)	0.40 (0.31)	0.45 ^§^ (0.44)
Serine	0.72 (0.43)	0.85 ^§^ (0.36)	0.91 ^§^ (0.40)	1.11 ^§^ (0.63)	1.11 (0.43)
Taurine	2.26 (2.65)	1.91 (1.78)	1.94 (1.31)	1.87 (1.42)	2.03 (1.12)
Tyrosine	0.38 (0.26)	0.40 (0.20)	0.28 ^§^ (0.17)	0.25 (0.14)	0.28 ^§^ (0.13)
**SUM**	20.1 (12.5)	25.5 ^§^ (10.4)	27.4 ^§^ (8.0)	29.0 (9.7)	28.6 (10.7)

^†^ IAA = indispensable amino acids; DAA = dispensable amino acid; ^‡^ Asx = sum of aspartic acid + asparagine; Glx = sum of glutamic acid + glutamine. Medians (inter-quartile ranges) of *n* = 90 samples per lactation period are shown. A median with a “^§^” superscript is significantly different from the median of the previous lactation period (*p* < 0.05).

## References

[1] Stam J., Sauer P.J., Boehm G. (2013). Can we define an infant's need from the composition of human milk?. Am. J. Clin. Nutr..

[2] Michaelsen K.F., Greer F.R. (2014). Protein needs early in life and long-term health. Am. J. Clin. Nutr..

[3] World Health Organization, Food and Agriculture Organization of the United Nations, United Nations University (2007). Joint FAO/WHO/UNU Expert Consultation on Protein and Amino Acid Requirements in Human Nutrition.

[4] Da Costa T.H., Haisma H., Wells J.C., Mander A.P., Whitehead R.G., Bluck L.J. (2010). How much human milk do infants consume? Data from 12 countries using a standardized stable isotope methodology. J. Nutr..

[5] Svanberg U., Gebre-Medhin M., Ljungqvist B., Olsson M. (1977). Breast milk composition in Ethiopian and Swedish mothers. III. Amino acids and other nitrogenous substances. Am. J. Clin. Nutr..

[6] Carratu B., Boniglia C., Scalise F., Ambruzzib A.M., Sanzinia E. (2003). Nitrogenous components of human milk: Non-protein nitrogen, true protein and free amino acids. Food Chem..

[7] Ventura A.K., Beauchamp G.K., Mennella J.A. (2012). Infant regulation of intake: The effect of free glutamate content in infant formulas. Am. J. Clin. Nutr..

[8] Zhang Z., Adelman A.S., Rai D., Boettcher J., Lőnnerdal B. (2013). Amino acid profiles in term and preterm human milk through lactation: A systematic review. Nutrients.

[9] Zhao X., Xu Z., Wang Y., Sun Y. (1989). Studies of the relation between the nutritional status of lactating mothers and milk composition as well as the milk intake and growth of their infants in Beijing. Pt. 4. The protein and amino acid content of breast milk. Acta Nutr. Sin..

[10] Ding M., Li W., Zhang Y., Wang X., Zhao A., Zhao X., Wang P., Sheng Q.H. (2010). Amino acid composition of lactating mothers' milk and confinement diet in rural North China. Asia Pac. J. Clin. Nutr..

[11] Yang T., Zhang Y., Ning Y., You L., Ma D., Zheng Y., Yang X., Li W., Wang J., Wang P. (2014). Breast milk macronutrient composition and the associated factors in urban Chinese mothers. Chin. Med. J. (Engl.).

[12] Blankenship D.T., Krivanek M.A., Ackermann B.L., Cardin A.D. (1989). High-sensitivity amino acid analysis by derivatization with O-phthalaldehyde and 9-fluorenylmethyl chloroformate using fluorescence detection: Applications in protein structure determination. Anal. Biochem..

[13] Lonnerdal B. (2003). Nutritional and physiologic significance of human milk proteins. Am. J. Clin. Nutr..

[14] Dupont C. (2003). Protein requirements during the first year of life. Am. J. Clin. Nutr..

[15] Hassiotou F., Geddes D.T. (2014). Programming of appetite control during breastfeeding as a preventative strategy against the obesity epidemic. J. Hum. Lac..

[16] Peace R.W., Gilani G.S. (2005). Chromatographic determination of amino acids in foods. J. AOAC Int..

[17] Affolter M., Garcia-Rodenas C.L., Vinyes-Pares G., Jenni R., Roggero I., Avanti-Nigro O., de Castro C.A., Zhao A., Zhang Y., Wang P. (2016). Temporal Changes of Protein Composition in Breast Milk of Chinese Urban Mothers and Impact of Caesarean Section Delivery. Nutrients.

[18] Sindayikengera S., Xia W.S. (2006). Nutritional evaluation of caseins and whey proteins and their hydrolysates from Protamex. J. Zhejiang Univ. Sci. B.

[19] Agostoni C., Carratu B., Boniglia C., Lammardo A.M., Riva E., Sanzini E. (2000). Free glutamine and glutamic acid increase in human milk through a three-month lactation period. J. Pediatr. Gastroenterol. Nutr..

[20] Reeds P.J. (2000). Dispensable and indispensable amino acids for humans. J. Nutr..

[21] Reeds P.J., Burrin D.G. (2001). Glutamine and the bowel. J. Nutr..

[22] Rezaei R., Wang W., Wu Z., Dai Z., Wang J., Wu G. (2013). Biochemical and physiological bases for utilization of dietary amino acids by young pigs. J. Anim. Sci. Biotechnol..

[23] Van der Hulst R.R., van Kreel B.K., von Meyenfeldt M.F., Brummer R.J., Arends J.W., Deutz N.E., Soeters P.B. (1993). Glutamine and the preservation of gut integrity. Lancet.

[24] Roig J.C., Meetze W.H., Auestad N., Jasionowski T., Veerman M., McMurray C.A., Neu J. (1996). Enteral glutamine supplementation for the very low birthweight infant: Plasma amino acid concentrations. J. Nutr..

[25] Burrin D.G., Stoll B. (2002). Key nutrients and growth factors for the neonatal gastrointestinal tract. Clin. Perinatol..

[26] Sánchez C.L., Cubero J., Sánchez J., Franco L., Rodríguez A.B., Rivero M., Barriga C. (2013). Evolution of the circadian profile of human milk amino acids during breastfeeding. J. Appl. Biomed..

